# The reference data for accuracy assessment of the Global Forest Watch tree cover 2000 in China

**DOI:** 10.1016/j.dib.2020.105238

**Published:** 2020-02-01

**Authors:** Di Zhang, Hao Wang, Xu Wang, Zhi Lü

**Affiliations:** School of Life Sciences, Peking University, Beijing, 100871, China

**Keywords:** Global forest watch, Tree cover 2000, Remote sensing, Forest cover, Accuracy, Reference data, Google earth, China

## Abstract

Remote-sensing products have emerged as key tools in forest cover monitoring. Their quality vary spatially, local validations are recommended before using the data for inventory and management tasks. We conducted a validation based on a visual interpretation procedure using high-resolution optical imagery on Google Earth to map the uncertainties and inaccuracies of Global Forest Watch (GFW) Tree Cover 2000 in China. The article provides the reference dataset applied in Zhang et al. (2020). The reference data has a total amount of 96 364 sample pixels collected using spatially stratified random sampling method. The samples were labelled with land use classifications and can provide further usage for remote sensing products.

**Table:** Specifications Table

Subject	Forestry
Specific subject area	Land Cover Analysis, Spatial Data Quality, Accuracy Analysis
Type of data	Table
How data were acquired	Instruments: software Visual interpretation of the high resolution images from Google Earth
Data format	Raw
Parameters for data collection	The GE images have relatively high resolution (>30m) and clear images that can allow a reliable classification into forest versus non-forest.
Description of data collection	We applied a spatially stratified random sampling strategy, and employed visual interpretation of the high resolution images from Google Earth (GE) to obtain reference classifications. The definition of forest in the reference data labelling was above 20% tree cover in each 30m*30m pixel. Images in the year 2000 were used as a priority, but for locations lacking images in 2000, we used the images from the nearest year in combination with the available time-series of images to assign the reference class.
Data source location	City/Town/Region: All regions in China Country: China Latitude and longitude (and GPS coordinates) for collected samples/data: longitude of E73°-E136° and the latitude of N17°-N55°
Data accessibility	Zhang, Di; Wang, Hao; Wang, Xu; Lu, Zhi (2020), “The Reference Data for Accuracy Assessment of the Global Forest Watch Tree Cover 2000 in China”, Mendeley Data, v1 https://doi.org/10.17632/3fg62njfw9.1
Related research article	Zhang, D., Wang, H., Wang, X. and Lü, Z. (2020) Accuracy assessment of the global forest watch tree cover 2000 in China. *International Journal of Applied Earth Observation and Geoinformation*, 87, pp. 102033.

**Table:** 

**Value of the Data** • The reference data with a large sample size provides a solid basis for evaluating the classification accuracy of forest remote sensing products. • The data can benefit researchers who use GFW products in their study, and looking for reference data to evaluate the accuracy of their remote sensing products. • The dataset can be used for land use study, as well as accuracy assessment of remote sensing products that contains the range of China. • The reference dataset provides geographical coordinates of the sample sites, land type information, and image year acquired on Google Earth.

## 1 Data description

This reference dataset sampled 96 364 pixels (30 m*30 m) in China for land use study. It was created to evaluate the classification accuracy of GFW Tree Cover 2000 in the range of China. The binary classifications are forest and non-forest. The definition of forest used in this dataset was tree stocked areas with a tree cover ≥20%, to be consistent with the definition we used in the GFW Tree Cover 2000. There are also sub-classifications of natural forest and plantation under the forest class natural forest and plantation under the forest class, and sub-classifications of water, farmland, deserts, grassland and buildings, etc. under the non-forest class. The labelling standard for sub-classifications referenced the classification standard of the current status of land use in the People's Republic of China (GBT21010-2007). We employed visual interpretation on Google Earth (GE) to label the reference samples. The error analysis and repeatability test show the dataset is reliable and in good quality to use (Zhang et al., 2020) [1]. In this dataset, we provided information including the geographical coordinates of the pixels (central points), land use classifications, and the earliest year of images used in GE. The reference dataset can serve for further usage in land use study and accuracy assessment of remote sensing products in the range of China.

## 2 Experimental design, materials, and methods

In sampling, we applied a spatially stratified random sampling strategy by dividing the sampling units into 1093 1° (altitude) × 1° (longitude) grid cells, and randomly selected 100 pixels from GFW Tree Cover 2000 in each grid. After dropped pixels fell out of the national boundary, the sample size of the dataset was 96 364 pixels.

In the labelling of reference samples, we employed visual interpretation of the high resolution images from GE to obtain reference classifications of forest and non-forest. The definition of forest in the reference samples was the same as used in the GFW (≥20% tree cover). We defined valid samples as pixels where the GE images have relatively high resolution and clear images that can allow reliable classification into forest versus non-forest. Images in the year 2000 were used as a priority, but for locations lacking images in 2000, we used the images from the nearest year in combination with the available time-series of images. Samples with resolution lower than 30 m or no reference images in GE were dropped from the dataset. This resulted in a number of 87 533 valid pixels in the dataset, and 70% (765 out of 1093) of the grid cells had more than 80 sample pixels.

In the classification labelling, the interpreters were asked to fill in the reference classifications while blinding the map category in the GFW to ensure independence. There were also sub-classifications of natural forest and plantation under the forest class, and sub-classifications of water, farmland, deserts, grassland and buildings, etc. under the non-forest class. We referenced the classification standard of the current status of land use in the People's Republic of China (GBT21010-2007) as the labelling standards. The classification protocol and images with reference labels can be found in the supplementary material of Zhang et al. [1].

When we collected the reference dataset, we did a repetition test to measure the quality of the reference classification as well as interpreter variability. The difference of accuracy results generated from three interpreters were all within 10%. The ground validation based on 777 random sample sites also showed an overall accuracy above 85% of the reference dataset. We hence assume, that our reference data is qualified to provide a reliable assessment of the GFW Tree Cover 2000.

## CRediT author statement

**Di Zhang**: Methodology, Data curation, Writing- Reviewing and Editing. **Hao Wang**: Conceptualization. **Xu Wang**: Data curation. **Zhi Lü**: Supervision.
